# Smart molecules: Serve today and make the future

**DOI:** 10.1002/smo.20230005

**Published:** 2023-06-19

**Authors:** Xiaojun Peng

**Affiliations:** ^1^ State Key Laboratory of Fine Chemicals Frontiers Science Center for Smart Materials Dalian University of Technology Dalian China; ^2^ State Key Laboratory of Fine Chemicals, College of Materials Science and Engineering Shenzhen University Shenzhen China

1

Molecule is a group of two or more atoms that form the smallest identifiable unit into which a pure substance can be divided and still retain the composition and chemical properties of that substance. Smart molecules are molecules or molecule‐based functional systems that show a response to external stimuli by means of a change of signal emission or action, such as light, heat, electric or magnetic fields, sound, stress, pH, moisture, guest molecules, et al.

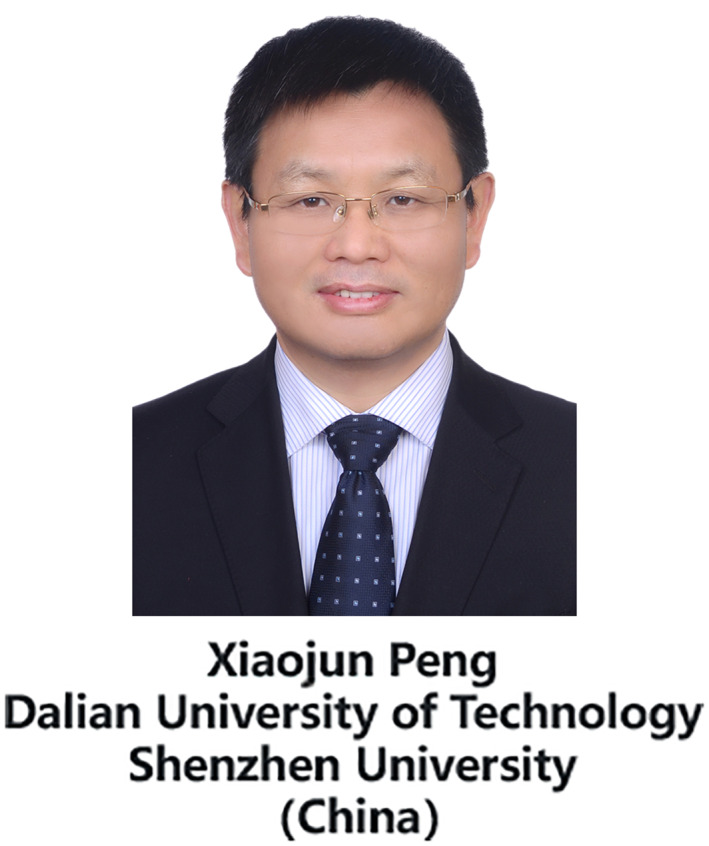

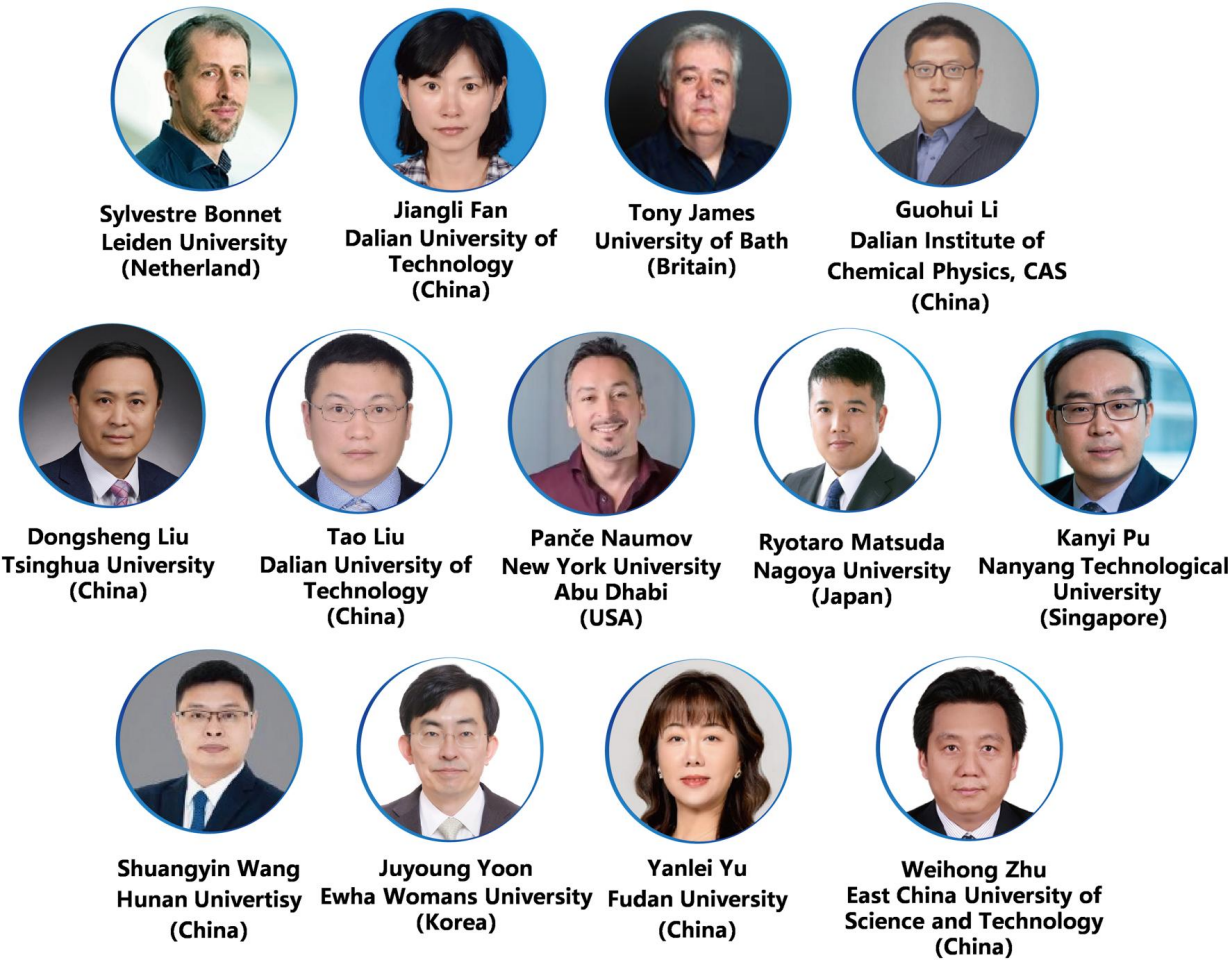

Smart molecules are advancing rapidly in life and health care. More recently, there has been a growing interest in “smart” molecules for a range of biomedical applications including precise drug delivery, diagnostics, tissue engineering and biomedical devices based on the communications and interactions between molecules and cells. As molecular machines, smart molecules can be controlled by external stimuli. Those features, combined with remarkable architectural versatility, make molecular machines uniquely powerful in a wide range of future technologies, from working as tiny robots for disease detection or drug delivery of microprocessors to specific sites.

Smart molecules also play key roles in clean energy science and technology. Smart molecule‐based artificial photosynthesis, integrated light‐harvesting antennae (photosensitizer), charge separation systems, and catalysts for water oxidation or hydrogen production based on covalently linked subunits are being elaborately designed and improved. In another respect, rapid progress in the development of organic photovoltaic (OPV) technology, numerous OPV materials including smart molecules have come to the fore in achieving outstanding power conversion efficiency and breaking 20% efficiency barrier in the single junction OPV devices. Innovative photovoltaic materials, especially molecular donors and acceptors, have become the dominant factor for improved device performance.

Smart molecules are widely used in digital displays. Now, fluorescent and phosphorescent organic dyes play an essential role in the creation of new “smart” molecular displays. Fragments and functional groups capable of free rotation around single bonds can significantly change the fluorescent and phosphorescent organic dye's electronic structure under analyte effects, phase state transitions, or changes in temperature, pressure, and media polarity. Effects of molecular packing of fluorescent and organic dyes are successfully used in developing mechano‐, piezo‐, and thermo‐fluorochromes materials in the optical recording of information, sensors, security items, memory elements, and organic light‐emitting diodes technologies.

As catalysis, smart molecules could not only promote chemical transformations including small organic molecules, molecular complexes, proteins and nucleic acids, but also act as reversible photoswitches for enantiospecific transformation and reversible photo‐superstructures, digital photoprogramming, and tunable circularly polarized luminescence with a high dissymmetry factor.

Recent years have witnessed the development of smart materials or stimuli‐responsive materials, which can sense the subtle variation in the environment and provide intrinsic property changes including shape, color, conductivity, light transmittance etc. Smart materials‐based actuators and robots are expected to be used in smart mechanical outputs, wearable and portable devices, bio‐inspired robots and surface haptics. Exploring these materials to construct “smart” mixed reality equipment will reduce the size and the weight of current devices as well as simplify the programming design to current control systems, offering better user experience.

*Smart Molecules* are copublished by Wiley and Dalian University of Technology and it aims to publish experimental and theoretical approaches dealing with molecules based functional systems that show a response to external stimuli, such as light, heat, electric field, magnetic field, sound, guest molecules, et al. It includes but not limited to the following topics: external stimuli induced cis‐trans, open‐close ring structural transformation, electric configuration interconversion such as spin transition and electron transfer detecting, sensing, self‐healing functions of molecules based functional systems, molecular devices and machine, micro and nano‐systems, key materials, mechanistic study, molecular design work, function tuning, smart manufacturing, et al.

*Smart Molecules* aims to publish a set of article types, such as research articles, reviews, perspectives, editorials and profiles. All articles accepted and published in *Smart Molecules* are fully Open Access: immediately freely available to read, download and share. A world‐class editorial group with scientists from different research backgrounds has teamed up for *Smart Molecules*. We proudly introduce our Editors‐in‐Chief, Prof. Xiaojun Peng (Dalian University of Technology, and Shenzhen University, China), our Associate Editors Prof. Sylvestre Bonnet (Leiden University, Leiden, Netherland), Prof. Jiangli Fan (Dalian University of Technology, Dalian, China), Prof. Tony James (University of Bath, Bath, UK), Prof. Guohui Li (Dalian Institute of Chemical Physics, Chinese Academy of Science, Dalian, China), Prof. Dongsheng Liu (Tsinghua University, Beijing, China), Prof. Tao Liu (Dalian University of Technology, Dalian, China), Prof. Ryotaro Matsuda (Nagoya University, Nagoya, Japan), Prof. Panče Naumov (New York University, New York, USA, and NYU Abu Dhabi, Abu Dhabi, United Arab Emirates), Prof. Kanyi Pu (Nanyang Technological University, Singapore), Prof. Shuangyin Wang (Hunan University, Changsha, China), Prof. Juyoung Yoon (Ewha Womans University, Seoul, Korea), Prof. Yanlei Yu (Fudan University, Shanghai, China), Prof. Weihong Zhu (East China University of Science and Technology, Shanghai, China). We believe that the world‐class editorial group will present our authors and reviewers with highly expert peer reviews and post‐acceptance processes. In addition, we sincerely thank the support from Dalian University of Technology and the professional guidance from Wiley.

We hope that our journal will provide multidisciplinary principles, broad scope, and high‐quality standards for a broad readership including chemists, physicists, materials scientists, nanotechnologists, engineers, biologists, medical researchers, and optoelectronic, environmental and analytical scientists from academia and industry as well as policy makers. We believe that *Smart Molecules* will develop quickly and become a leading open‐access journal in the near future.

## CONFLICT OF INTEREST STATEMENT

The authors declare no conflicts of interest.

